# Clustering and Characteristics of Acute Acquired Brain Injury Patients With Driving Resumption Difficulty: The Role of Neuropsychological Tests, Frailty, and Gait Independence

**DOI:** 10.7759/cureus.80735

**Published:** 2025-03-17

**Authors:** Eisei Harayama, Yuta Miyahara, Shota Tanaka, Kota Yamauchi, Masato Osaki, Shuji Arakawa

**Affiliations:** 1 Department of Rehabilitation, Steel Memorial Yawata Hospital, Kitakyushu, JPN; 2 Department of Behavior and Health Sciences, Kyushu University, Fukuoka, JPN; 3 Department of Stroke and Neurological Center, Steel Memorial Yawata Hospital, Kitakyushu, JPN

**Keywords:** acute brain injury, cluster analysis, frailty, resuming driving, walking ability

## Abstract

Objectives: Decisions to resume driving after acute brain injury are often difficult. Predictors of decisions depend on the results of neuropsychological tests. As a result, few studies have investigated other characteristics. This study clustered participants who received support for resuming driving after acute brain injury based on neuropsychological tests and analyzed their characteristics.

Materials and methods: Participants were 74 patients with acute brain injury. Cluster analysis was used to classify participants based on attention and visuospatial cognitive functions. Each cluster group were compared using multiple comparison tests (Bonferroni method) in terms of clinical assessments, neuropsychological tests, and driving resumption ability.

Results: The characteristics of the cluster group with poor results in the two neuropsychological tests were that they were often judged as unable to resume driving (p < 0.001), were in a state of frailty (p = 0.02), and had a low level of walking independence (p = 0.02).

Conclusions: When helping patients with acute brain injury to return to driving, not only the results of neuropsychological tests but also assessments of frailty before onset and level of walking independence are important.

## Introduction

Driving is an essential mode of transportation. In recent years, there has been an increasing need for patients with acquired brain injuries (ABI), such as stroke or traumatic brain injury (TBI), to resume driving. Neuropsychological tests for evaluating the driving ability of stroke patients are classified into four categories: executive function, perception/cognition, attention/memory, and language [1]. Similarly, executive function, verbal memory, speed/attention processing, and visual memory are crucial for driving ability in patients with TBI [2]. Several predictive measures of return to driving after ABI have been validated, including the Mini-Mental State Examination (MMSE) [3], Trail Making Test (TMT) [4], and the Rey-Osterrieth Complex Figure (ROCF) [5]. Among intelligence, attention, visuospatial cognition, memory, executive function, language, attention, and visuospatial cognition are particularly crucial for on-road driving [6], and the TMT Part B and ROCF are considered useful predictors of driving skills and abilities [1,7].

In general, the support and assessment of return to driving after an ABI are performed by combining neuropsychological testing and driving simulator assessments before the actual vehicle evaluation, called off-road evaluation [8], and on-road evaluation, where the patient actually drives a car and is assessed [9]. However, a reliable support method is yet to be developed. In clinical practice, cases have been studied where off-road evaluation is considered unproblematic; however, the actual on-road evaluation does not allow the patient to resume driving. Conversely, cases exist where off-road evaluation is problematic and a strong impact on driving is predicted; however, the on-road evaluation is unproblematic. These discrepancies make clinical judgment difficult. The two should be combined for an appropriate judgment. However, limitations exist to judging whether driving can be resumed based on clinical neuropsychological tests. Particularly in Japan, which has become a super-aging society, it is necessary to provide driving support by considering the multifaceted factors of aging.

It is desirable to make a decision based on the results of neuropsychological testing, including physical function, motor ability, and degree of independence in activities of daily living (ADL) after ABI. Frailty [10], a state of increased mental and physical vulnerability to external stress due to different functional changes and a decline in physiological reserve capacity associated with aging, may affect the decision to resume driving, but this has not been fully verified. Although it is meaningful to use logistic regression analysis to determine the relative risk of factors affecting whether or not driving can be resumed after ABI, when the sample size of subjects is small, there is a possibility that the accuracy of the model may be questionable. Therefore, we investigated a method called cluster analysis. We clustered those who were supported in resuming driving after an ABI based on neuropsychological testing and hypothesized that those in the poor off-road assessment group would have difficulty resuming driving, have frailty before the onset of the disease, and have lower functional ability after the onset of the disease compared with those in the good off-road assessment group. The purpose of this study was to cluster patients who were supported in resuming driving after an ABI based on neuropsychological testing and to analyze their characteristics.

## Materials and methods

Study design

This study was a single-center, retrospective, observational study.

Participants and setting

The study was conducted between June 2019 and March 2024. The subjects were patients with ABI who were treated for stroke or TBI at our hospital. Stroke refers to patients with cerebral hemorrhage and cerebral infarction, and TBI refers to mild cases of chronic subdural hematoma surgery. Among the patients with ABI who were discharged, those who had the need and desire to resume driving to return home after discharge and those who were supported to resume driving were studied. The inclusion criteria were as follows: 1) ABI patients aged 65 years or older, 2) those who wanted to resume driving after discharge and were supported to do so, and 3) those who understood and consented to this study. The study was conducted in accordance with the principles of the Declaration of Helsinki. The purpose of the study and ethical considerations were adequately explained to the participants. Appropriate measures were implemented to protect their personal information, and informed consent was obtained. This study was approved by the Ethics Committee of Steel Memorial Yawata Hospital (approval number: 24-53).

Outcome/event

The ABI support for resuming driving is provided in three stages: off-road assessment at a medical institution, on-road assessment at a designated driving school, and approval by the National Public Safety Commission. Step 1, the off-road assessment, explains the content of the support and the procedure required to resume driving. Physical function and motor ability were assessed by a physical therapist, and neuropsychological testing was performed by an occupational therapist. Aphasia and other conditions were assessed by a speech therapist when necessary. After each assessment, the attending physician was informed of the underlying disease and that there was no risk of resuming driving. The second stage of the on-road assessment was performed by a trained instructor at a designated driving school, and an actual vehicle was assessed. Step 2 was implemented and assessed after patients were discharged from the hospital. Finally, based on the results of the off-road and on-road assessments, the third stage was determined using an aptitude test by the Public Safety Commission [11]. In the off-road assessment, those who are judged to have little difficulty driving based on the results of the neuropsychological test may be permitted in the first stage. However, the decision to resume driving is typically made in three stages.

Measurements

Age, sex, body mass index (BMI), diagnosis, site of injury, length of hospital stay, frailty before onset, modified Rankin Scale (mRS) score, National Institutes of Health Stroke Scale (NIHSS) score, and polypharmacy were assessed. The presence or absence of frailty was determined at the time of admission using the Kihon Checklist (KCL), and the state of frailty before the onset was assessed. The KCL was developed by the Ministry of Health, Labour and Welfare of Japan as a comprehensive scale to identify older adults at risk of functional disability in the near future [12]. Moreover, it has been validated against the cardiovascular health study (CHS) used in other countries [13]. Responses to the KCL were considered robust if the total score of the 25 items was 3 or less, pre-frailty if it was 4-7, and frailty if it was 8 or more [14]. The mRS is a general prognostic assessment scale for stroke and other conditions and is an index score on a 7-point scale from grade 0 (asymptomatic) to grade 6 (death). We used it as a functional index before hospitalization. NIHSS is an index that assesses the severity of stroke and is scored from 0 to 42 points, with higher scores indicating higher severity [15]. Polypharmacy was defined as the use of five or more medications per day [16]. All of these items were assessed at the time of admission.

Clinical assessments

We examined whether a history of falls existed during hospitalization and the motor items of the Stroke Impairment Assessment Set (SIAS) were used to assess functional impairment, grip strength (kg) on the unaffected side, walking speed (m/s), and functional ambulation categories (FAC). These items were assessed by physical and occupational therapists before discharge. A history of falls during hospitalization was defined by Gibson [17] as “involuntary contact of any part of the body with the ground or a lower surface,” and a history of falls was assessed if a fall or slip was observed during the period of inpatient treatment for ABI. The SIAS is a comprehensive functional assessment that assesses the motor function of the paretic side on a scale of 0-5 and the muscle tone, sensation, range of motion, pain, trunk function, higher brain function, and function of the non-paralyzed side on a scale of 0-3 [18]. The SIAS-m was assessed using the paralyzed-side motor items of the SIAS. The grip strength of the unaffected side was measured twice using a digital grip strength meter (Takei; TKK-5401, Japan), and the average value was calculated. Walking speed was measured twice using a digital stopwatch (CASIO; HS-3C-8AJH) for a maximum walking distance of 10 m, and the average value was calculated. The walking distance was set at 10 m on floor level. The FAC [19] is an index that classifies walking ability into six levels of independence based on the amount of physical assistance required, and FAC ≥ 4 was defined as walking independence. ADL was assessed using the Barthel index (BI), with a BI of >80 defined as independent and a BI of <80 defined as non-independent [20].

Neuropsychological tests were performed to assess the following: the cognitive function was measured by the score on the Japanese version of the MMSE [21], attention function was measured by the time required to complete the Japanese version of the TMT Part B (TMT B) [22], frontal lobe function was measured by the score on the Frontal Assessment Battery (FAB) [23], and visuospatial cognitive function was measured by the score on the ROCF [24]. Neuropsychological tests were assessed before discharge.

Statistical analysis

For statistical analysis, the participants were clustered using hierarchical cluster analysis (Ward’s method). The variables used for clustering were the TMT B and ROCF, and the groups were categorized. These variables were selected because TMT B and ROCF, which are related to driving skill prediction and assessments, are useful for driving skill prediction [7]; therefore, they were used as the clustering variables in this study. Because the scoring method differed depending on the evaluation item, the TMT B and ROCF scores were converted to standardized scores (Z-value conversion) when performing the cluster analysis.

The Shapiro-Wilk test was used to confirm the normal distribution of each variable. The test revealed that BMI, grip strength, and walking speed followed a normal distribution. Between-group comparisons were performed to analyze the characteristics of each group categorized by cluster analysis. The assessment items of each group were compared using the chi-squared test for nominal variables, the Kruskal-Wallis test for ordinal variables, and one-way analysis of variance (ANOVA) for continuous variables. In addition, multiple comparison tests using the Bonferroni method were performed for all between-group comparisons. For participants with missing data (n = 5), the variables were replaced with the mean using mean substitution [25]. Statistical analyses were performed using SPSS for Windows (version 23; IBM Japan, Japan), and the significance level was set at 5% for all cases.

## Results

**Figure 1:**
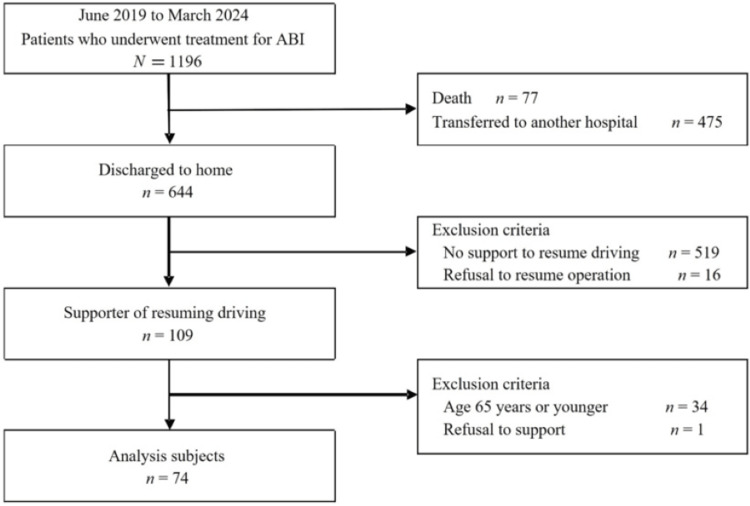
Flowchart of the study. ABI: acquired brain injuries

Of the 1,196 patients treated at our hospital between June 2019 and March 2024, 643 were discharged, excluding deaths (n = 77) and transfers (n = 476). Of these, 109 patients who received assistance in resuming driving were included in this study. Furthermore, patients faged < 65 years and those who refused driving resumption assistance were excluded, leaving 74 patients who met the eligibility criteria and were included in the analysis (Figure 1).

Basic attributes of subjects

**Figure 2:**
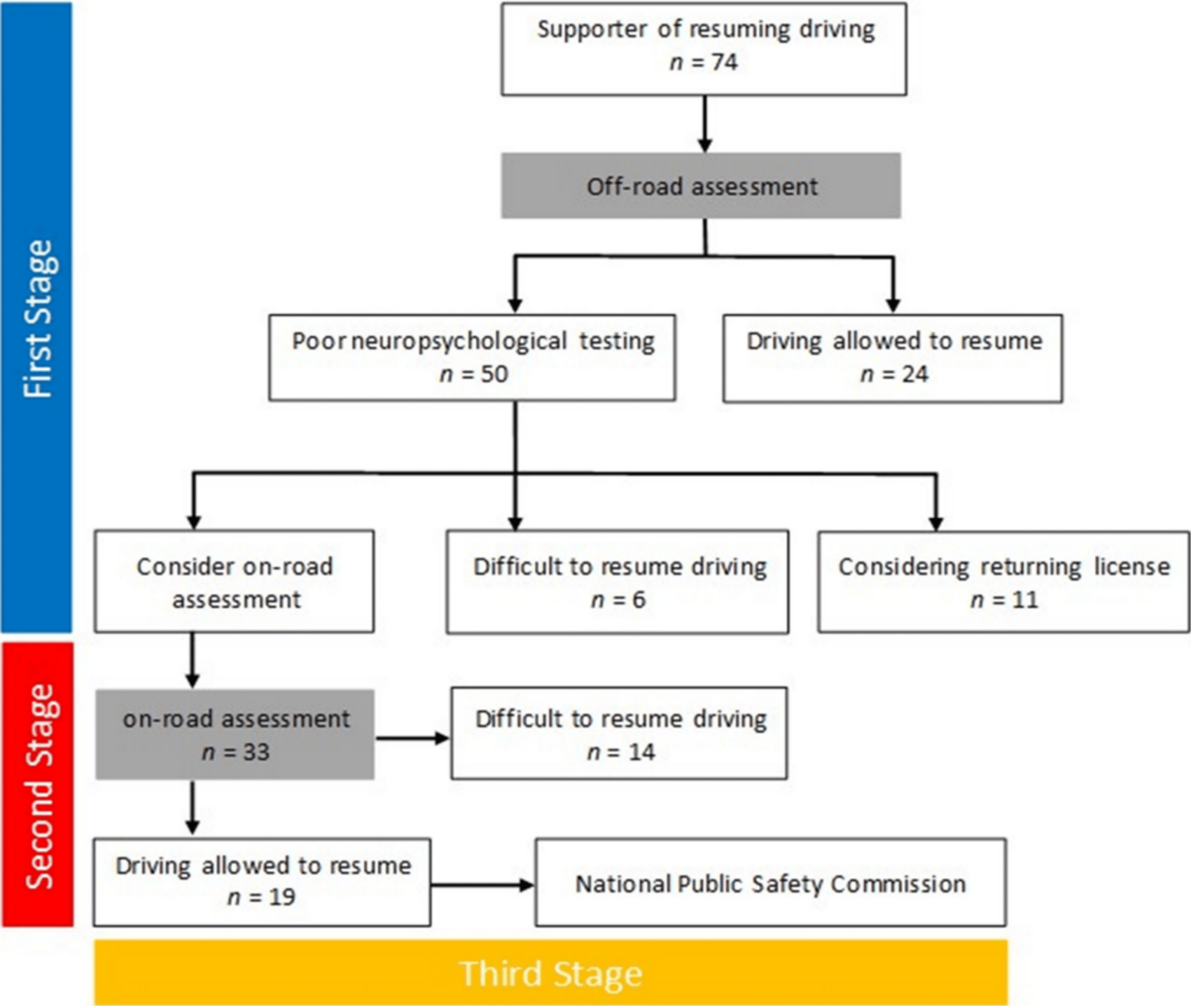
Flowchart of study results on whether or not to resume driving.

The subjects were aged 75.0 ± 5.9 years, 61 males (82%), BMI 23.5 ± 3.2 kg/m^2^, 60 diagnosed with cerebral infarction (81%), eight with cerebral hemorrhage (11%), and two with chronic subdural hematoma (8%). The length of hospital stay was 23.7 ± 15.2 days. Before disease onset, the mRS score was 0, the NIHSS score was 2, and 55 patients (74%) were taking multiple medications. Before the onset, 20 patients (27%) had frailty, 26 (35%) had pre-frailty, and 28 patients (38%) had robust symptoms. Eleven patients (15%) had a history of falls during hospitalization, the SIAS-m score was 23.9 ± 1.8 points, the grip strength on the healthy side was 27.1 ± 7.3 kg, the walking speed was 1.0 ± 0.2 m/s, and FAC ≥ category 4 was 93%. The MMSE score was 27.2 ± 3.0, and the FAB score was 14.6 ± 2.5 (Table 1). Regarding whether or not patients were able to resume driving after receiving assistance, 43 patients (58%) patients were able to resume driving after receiving assistance (Figure 2). Twenty-four patients were allowed to resume driving after the in-hospital evaluation, and 19 patients were allowed to resume driving after the on-road evaluation. The details of patients who were unable to resume driving are presented in Table 2.

**Table 1:** Patient basic characteristics. Continuous variables are presented as mean ± SD. Categorical variables are presented as numbers (%). Continuous variables are presented as median. BMI: body mass index; LOS: length of stay; mRS: modified Rankin scale; NIHSS: National Institutes of Health Stroke Scale; KCL: Kihon checklist; SIAS-m: Stroke Impairment Assessment Set-motor; BI: Barthel index; FAC: Functional Ambulation Categories; MMSE: Mini Mental State Examination; TMT B: Trail Making Test Part B; FAB: Frontal Assessment Battery; ROCF: Rey-Osterrieth Complex Figure; SD: standard deviation

Items	All subjects (n=74)
Age (years)	75.0 ± 5.9
Male Sex (%)	61 (82)
BMI (kg/m^2^)	23.5 ± 3.2
Diagnosis n (%)	
Cerebral infarction	60 (81)
Cerebral hemorrhage	8 (11)
Chronic subdural hematoma (after treatment)	2 (8)
Affected hemisphere n (%)	
Right hemisphere	36 (49)
Left hemisphere	34 (46)
Both hemispheres	4 (5)
LOS (days)	23.7 ± 15.2
mRS (category)	0 (0-0)
NIHSS (points)	2 (1-4)
Polypharmacy yes (%)	55 (74)
KCL (points)	
Frailty (≥8)	20 (27)
Prefrailty (4-7)	26 (35)
Robust (≤3)	28 (38)
History of falls, yes (%)	11 (15)
SIAS-m, (points)	23.9 ± 1.8
Grip strength (kg)	27.1 ± 7.3
Walking speed, (m/s)	1.0 ± 0.2
BI (>80 points), yes (%)	73 (99)
FAC (≥category 4), yes (%)	69 (93)
MMSE (points)	27.2 ± 3.0
TMT B (sec)	188.3 ± 120.6
FAB (points)	14.6 ± 2.5
ROCF (points)	31.5 ± 4.8
Final decision on resuming driving, yes (%)	43 (58)

**Table 2:** Details of the doctor's decision not to resume driving.

Promise not to resume driving	Number of subjects (n = 31)	Reason
Considering voluntary return	n = 11	As a result of the support, he consulted with his doctor and family and returned his license.
Higher brain dysfunction	n = 12	It was determined that he had poor awareness of his illness, attention disorder, and visual-spatial cognitive impairment, which would impede his ability to drive.
Cognitive impairment	n = 2	It was determined that he would be unable to make appropriate driving decisions.
Disease management	n = 6	There were concerns that he would be unable to drive due to the need for continued treatment for complications (cancer, heart disease, risk of recurrence).

Cluster analysis of TMT B and ROCF

**Figure 3:**
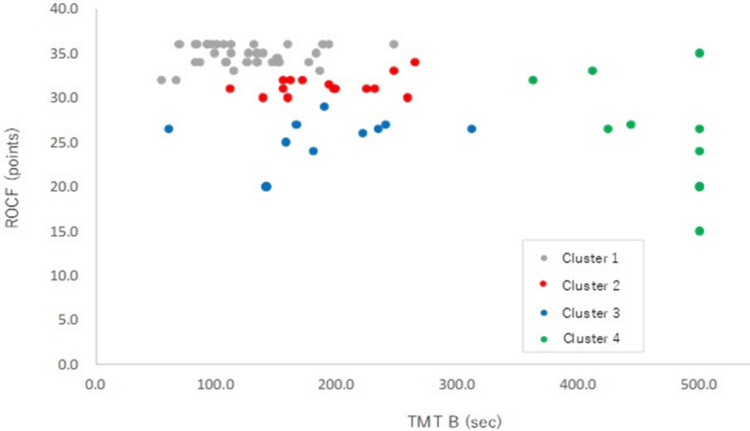
Scatter plot showing the TMT B performance time and ROCF scores. ROCF: Rey–Osterrieth Complex Figure; TMT B: Trail Making Test Prat B

Using hierarchical cluster analysis, supporters of driving restarts were grouped into four clusters. Group typology by cluster analysis was classified as Cluster 1 (n = 38) with good TMT B and ROCF, Cluster 2 (n = 15) with good ROCF score and delayed TMT B, Cluster 3 (n = 11) with no delay in TMT B and poor ROCF score, and Cluster 4 (n = 10) with delayed TMT B and poor ROCF score (Table 3). A scatterplot of the driving restart supporters’ TMT B performance time and ROCF score is shown in Figure 3.

**Table 3:** Classification of populations using hierarchical cluster analysis. ^1^n (%), mean (standard deviation) TMT B: Trail Making Test Part B; ROCF: Rey-Osterrieth Complex Figure

Total n=74	Cluster 1 n=38^1^	Cluster 2 n=15^1^	Cluster 3 n=11^1^	Cluster 4 n=10^1^
TMT B (sec)	124.7 ± 40.4	190.8 ± 44.6	185.5 ± 62.7	464.0 ± 48.0
ROCF (points)	34.8 ± 1.2	31.4 ± 1.1	25.2 ± 2.7	25.9 ± 6.0

Comparison of patient characteristics by group classification

Patient characteristics for each cluster and multiple comparison tests (Bonferroni method) showed a significant difference in the presence or absence of frailty when comparing cluster 4 with clusters 1 and 2 (p = 0.02) (Table 4). In addition, cluster 4 had lower walking independence based on FAC (p = 0.02), lower cognitive function (p < 0.001), and lower frontal lobe function (p < 0.001) than cluster 1, and cluster 4 was more likely than cluster 1 to decide that driving could not resume (p < 0.001) (Table 4).

**Table 4:** Characteristics of each cluster and patient background and multiple comparison tests. ^1^Kruskal-Wallis test;^ 2^Chi-square tests; ^3^ANOVA BMI: body mass index; LOS: length of stay; mRS: modified Rankin Scale; NIHSS: National Institutes of Health Stroke Scale; KCL: Kihon checklist; SIAS-m: Stroke Impairment Assessment Set-motor; BI: Barthel index; FAC: Functional Ambulation Categories; MMSE: Mini Mental State Examination; FAB: Frontal Assessment Battery; ANOVA: Analysis of Variance.

Variable	Cluster 1, n=38	Cluster 2, n=15	Cluster 3, n=11	Cluster 4, n=10	P-value	Bonferroni
Age (years)	73.5 ± 5.6	75.7 ± 6.1	77.3 ± 6.9	77.1 ± 3.8	0.15^1^	
Male sex n (%)	31 (86)	11 (73)	11 (100)	9 (90)	0.35^2^	
BMI (kg/m^2^)	23.5 ± 2.6	22.2 ± 3.9	23.8 ± 2.3	25.4 ± 3.9	0.32^3^	
LOS (days)	19.3 ± 11.0	28.5 ± 14.1	22.5 ± 10.1	34.2 ± 24.5	0.06^1^	
mRS	0 (0-0)	0 (0-1)	0 (0-0)	0 (0-1)	0.90^1^	
NIHSS	1 (1-4)	3 (2-6)	4 (1-6)	3 (1-5)	0.26^1^	
Polypharmacy, yes (%)	26 (68)	12 (80)	8 (73)	9 (90)	0.52^2^	
KCL (points)					0.02^2^	
Frailty (≥8)	6 (16)	3 (20)	5 (45)	6 (60)		1>4, 2>4
Prefrailty (4-7)	17 (45)	7 (47)	0 (0)	2 (20)		1>3, 2>3
Robust (≤3)	15 (39)	5 (33)	6 (55)	2 (20)		No significant difference
History of falls, yes (%)	3 (9)	2 (13)	2 (18)	4 (40)	0.09^2^	
SIAS-m (points)	24.3 ± 1.4	23.3 ± 2.3	23.8 ± 2.0	23.9 ± 2.0	0.69^1^	
Grip strength (kg)	28.6 ± 7.7	26.9 ± 7.4	26.8 ± 3.6	22.1 ± 6.1	0.10^3^	
Walking speed (m/s)	1.1 ± 0.2	1.0 ± 0.2	1.0 ± 0.3	0.9 ± 0.3	0.15^1^	
BI (>80 points), yes (%)	38 (100)	15 (100)	11 (100)	9 (90)	0.02^2^	1, 2, 3>4
FAC (≥ category 4), yes (%)	38 (97)	15 (100)	11 (100)	6 (60)	0.003^2^	1, 2, 3>4
MMSE (points)	28.2 ± 2.4	26.6 ± 2.8	27.7 ± 1.8	23.8 ± 3.7	0.001^1^	1>4, 1>2, 3>4
FAB (points)	16.0 ± 1.6	13.1 ± 2.6	14.2 ± 2.0	12.3 ± 2.4	<0.001^1^	1>2, 1>3, 1>4
Final decision on resuming driving, yes (%)	32 (84)	7 (47)	4 (36)	1 (10)	<0.001^2^	1>2, 3, 4

## Discussion

In this study, driving resumption supporters for patients with ABI were categorized into clusters based on neuropsychological tests using the TMT B and ROCF, and the characteristics of the patients in each cluster were analyzed. Supporters of driving resumption were categorized into four clusters, and comparisons were made between the groups. Cluster 4, categorized as having a delayed performance time on TMT B and poor ROCF scores, had more frailty than the other clusters, lower walking independence, impaired cognitive function, and frontal lobe function and was more likely to be judged as unable to resume driving after driving resumption support.

Neuropsychological testing and ability to resume driving

ABI (cluster 4), which was categorized as poor performance time on TMT B and poor ROCF score, was more likely to be judged as unable to drive when deciding whether to resume driving. In general, attention and visual-spatial cognitive functions are important in road driving [7], and TMT B and ROCF are useful predictors of driving skills and driving ability [1,7]. Therefore, this study supports previous studies. In addition, this study was characterized by a decline in cognitive and frontal lobe functions. Drivers aged 65 years or older with a history of driving accidents have lower cognitive function (including attention) than those without a history of accidents [26,27]. A decline in cognitive function [3] is a useful predictor of driving resumption in stroke patients. Although the characteristics of cluster 4 were in the non-dementia area, a significant difference compared to the other clusters was noted. Therefore, the MMSE is an important evaluation index for resuming driving. In this study, the FAB level was also an important indicator. In general, the FAB assesses frontal lobe function and consists of six items: word conceptualization, verbal fluency, motor programming, susceptibility to interference, inhibitory control, and comprehension behavior [23]. Because several patients in cluster 4 had frontal lobe dysfunction, it is important to independently assess frontal lobe function in patients with ABI who have attentional or visuospatial cognitive dysfunction. Recent studies have reported that FAB is also an indicator for determining whether a patient can resume driving [11], and further verification is required.

Before onset frailty

In this study, pre-onset frailty was measured using the KCL, which consisted of 25 questions. KCL is a comprehensive scale developed by the Ministry of Health, Labour and Welfare of Japan to identify elderly people at risk of functional impairment in the near future [12]. Therefore, although it is not a direct assessment index of frailty, it has a high positive correlation with frailty as determined by the CHS, and its validity has been demonstrated [14]. Cluster 4 had the highest rate of frailty. In recent years, frailty has been considered in relation to driving accidents among community-dwelling older adults [28,29]. Although it is unclear whether frailty is a direct factor in the inability to resume driving after ABI, we believe that the assessment of frailty before onset is important. In addition to the frailty state associated with aging, resuming driving may be difficult owing to the disease-specific higher brain dysfunction and physical function decline caused by ABI.

FAC After ABI

Cluster 4 had low walking independence as assessed by the FAC after the onset of ABI. FAC is an index that assesses the degree of walking independence on a 6-point scale based on the amount of physical assistance required [19]. FAC ≤ 3 is considered to be non-walking independent, and FAC ≥ 4 is considered to be walking independent [19]. The subjects in this study had a SIAS-m score of 23.9 ± 1.8 points, which is less affected by motor paralysis. Therefore, higher brain dysfunction due to ABI might have had an effect. Attention disorders are characteristic of higher brain dysfunction after an ABI. Attention functions that can be assessed by the TMT are divided into the following four categories: (i) cognitive processing speed required for rapid task completion, (ii) sustained attention during continuous task performance, (iii) visual search ability based on spatial attention, and (iv) working memory, divided attention. Attention disorders due to these factors are likely to affect walking status. In addition, ROCF, a visuospatial cognitive function marker, may affect FAC. The ROCF is a standardized assessment to measure visuospatial cognition, structuring function, and non-verbal visual memory [24]. In the context of driving, it is also an integral part of visuospatial cognition, information processing, and short-term memory of visual data obtained by scanning one’s own or others’ vehicles [6]; it is possible that these situations are also common to walking situations. Cluster 4 had a low degree of walking independence based on the FAC; therefore, the assessment of walking ability is important.

Strengths and limitations

This study had several limitations. First, this was a single-center study in an acute care hospital, and the sample size was small. Therefore, the design of this study did not allow for adjustment for confounding factors by multivariate analysis. In addition, this study did not examine the predictors of difficulty in returning to driving, a cause-and-effect relationship could not be established. Additionally, this study did not include patients with moderate-to-severe ABI who required a transfer to a rehabilitation hospital. In the future, it will be necessary to verify a larger number of cases. Second, in this study, we were unable to assess MOCA, which can better assess attention and visuospatial cognitive functions. It is possible that different results could be obtained by analysis using MOCA. Third, for missing data (n = 5), the mean substitution of variables was replaced with the mean value. Therefore, the possibility of a data bias cannot be excluded. Fourth, the study included patients who wanted to drive but were unable to do so for medical reasons, such as the risk of relapse or seizures. In addition, cases of polypharmacy with psychotropic medication were not included. Although we sought a physician's opinion, we may not have been able to completely exclude cases taking medications that may have some effect on driving. Fifth, the on-road assessment was limited to designated driving schools. However, the possibility of bias between facilities and evaluators cannot be completely excluded. The results should therefore be interpreted with caution. Finally, although some neuropsychological tests may be confounding factors in driving resumption after ABI, other true confounding factors remain unknown. Further research is needed.

## Conclusions

We conducted a cluster analysis to classify patients using two neuropsychological tests: TMT B and ROCF. Cluster groups with poor scores on the two neuropsychological tests were more likely to be judged as unable to resume driving, to be in a state of frailty, to have low levels of walking independence, and to have impaired cognitive and frontal lobe functions.

Therefore, it is important to assess patients' cognitive and frontal lobe functions when assessing whether or not they can resume driving after ABI. In addition, it is necessary to pay attention to frailty before onset and walking ability after ABI.
